# Influence of glutathione S‐transferases (GSTM1, GSTT1, and GSTP1) genetic polymorphisms and smoking on susceptibility risk of chronic myeloid leukemia and treatment response

**DOI:** 10.1002/mgg3.717

**Published:** 2019-05-20

**Authors:** Golale Rostami, Dlnya Assad, Fatemeh Ghadyani, Mohammad Hamid, Amirhossien Karami, Hasan Jalaeikhoo, Ramezan Ali Kalahroodi

**Affiliations:** ^1^ Department of Molecular Medicine, Biotechnology Research Center Pasteur Institute of Iran Tehran Iran; ^2^ Department of Biology, College of Science Sulaimani University Sulaymanyah Iraq; ^3^ Department of Cellular and Molecular, Faculty of Biology Sciences Islamic azad university of Tehran North Tehran Iran; ^4^ AJA Cancer Epidemiology Research and Treatment Center (AJA‐ CERTC) AJA University of Medical Sciences Tehran Iran; ^5^ AJA Cancer Research Center (ACRC) AJA University of Medical Science Tehran Iran

**Keywords:** chronic myeloid leukemia, genetic polymorphisms, glutathione S‐transferases, smoking status, treatment responses

## Abstract

**Background:**

Glutathione S‐transferases (GSTs) polymorphisms may impact on chronic myeloid leukemia (CML) risk or heterogeneous responses to Imatinib mesylate (IM). The aim of this study was to evaluate the correlation between GSTs polymorphisms and CML risk, treatment response.

**Methods:**

We genotyped GSTM1, GSTT1 null deletion polymorphisms, and GSTP1 Ile105Val polymorphism by PCR methods and BCR‐ABL transcripts were analyzed by qRT‐PCR in 104 CML patients and 104 sex‐ and age‐matched healthy individuals.

**Results:**

Individual analysis showed significant association of GSTM1 (*p* = 0.008; OR = 0.46; 95% CI: 0.26–0.82) and GSTP1 genes (*p* = 0.04; OR = 1.56; 95% CI: 1.016–2.423) with CML risk. The combined analysis indicated that GSTM1 null/GSTT1 present, GSTM1‐null/GSTP1M*(AG/GG) as well as GSTT1 present/ GSTP1M* genotype were associated with CML risk (ORg(‐):2.28; 95% CI: 1.29–4.04; ORgg: 2.85; 95% CI: 1.36–5.97; OR(‐)g: 1.75; 95% CI: 0.99–3.06, respectively). The proportion of CML cancer attributable to the interaction of smoking and GSTM1 null, GSTT1null, and GSTP1 M* was 42%, 39%, and 13%, respectively. Patients with GSTM1‐null and GSTP1 AG/GG genotype had significantly a lower rate of MMR achievement (*p* = 0.00; *p* = 0.009 respectively). Event‐free survival (EFS) percentage was similar between GSTM1 null and GSTM1 present patients (*p* = 0.21).

**Conclusion:**

Our study suggests the influence of GSTM1 and GSTP1 polymorphisms on CML risk and treatment response. The interaction between GSTs polymorphisms and smoking plays a significant role on CML susceptibility.

## 1 INTRODUCTION

Chronic myeloid leukemia (CML) is a myeloproliferative disease and an outcome of the reciprocal translocation t (9;22) (q34;q11) or the BCR‐ABL1 fusion gene (Kassogue et al., 2014; Rostami, Hamid, Yaran, Khani, & Karimipoor, 2015). The tyrosine kinase inhibitor (TKI), Imatinib mesylate (IM) is a potent Bcr‐Abl1–targeting drug with remarkable clinical benefits. Despite the excellent results achieved with IM regarding to the improvement of quality of life and patient survival, almost 40% of CML patients will finally fail IM treatment and demand second‐generation (2G) TKIs such as nilotinib or dasatinib (Davies et al., 2014; Francis et al., 2013). However, treatment failure to 2G TKIs as initial therapy due to toxicity has been reported (Eghtedar et al., 2013). Development of resistance to TKIs in CML has been assigned to Bcr‐Abl‐dependent and ‐independent mechanisms, and mutations of the ABL1 kinase domain are the most general cause of resistance (Rostami et al., 2015). It has also been reported that polymorphism in genes associated to drug bioavailability and metabolizing enzymes may affect treatment outcome (Weich et al., 2016). Earlier association studies have identified the roles of polymorphic variants in different crucial genes associated with predisposing individuals to CML, with incompatible results (Bruzzoni‐Giovanelli et al., 2015; He et al., 2014). These incompatibilities may be due to lifestyle factors, ethnic differences, environmental exposures in population cohorts studied (Davies et al., 2014; Özten, Sunguroğlu, & Bosland, 2012). Glutathione S‐transferase (GST) genes, an important family of phase II metabolizing enzymes, catalyze the conjugation of a large spectrum of endogenous and exogenous compounds, including carcinogens, anticancer drugs, and their metabolites with reduced glutathione, thus enabling their detoxification and elimination (Hayes, Flanagan, & Jowsey, 2005). Detoxification of activated carcinogens by hydrolysis, reduction or oxidation protects the cells from producing of DNA adducts, genomic instability, and finally the initiation of cancer (Dusinska et al., 2012; Laborde, 2010). It has been suggested that individual inherited genetic differences in the ability to metabolize carcinogens correlated with polymorphism in detoxification enzymes may influence in carcinogen metabolism and cancer susceptibility (Taspinar et al., 2008; Yaya et al., 2014). Whole‐gene deletion polymorphisms of GSTM1 (OMIM accession number: *138350) and GSTT1 (OMIM accession number:*600436) genes and the single nucleotide polymorphism (SNP) in GSTP1 c. 313 A>G ( p. 105Ile>Val; rs1695) (OMIM accession number:*134660) lead to the absence or decreased detoxification ability of enzymes, their dysfunction, and finally may impact on the risk of cancer development, heterogeneous drug responsiveness (Davies et al., 2014; Hollman, Tchounwou, & Huang, 2016; Özten et al., 2012). Several studies determined only the role of GST polymorphisms on CML susceptibility (Al‐Achkar, Azeiz, Moassass, & Wafa, 2014; He et al., 2014; Özten et al., 2012) and some studies assessed their impact on treatment response (Davies et al., 2014; Kassogue et al., 2014). A recent study has evaluated the effect of GST polymorphisms on CML susceptibility and treatment response, but the interaction between these genes and environmental exposures or risk factors such as cigarette smoke was not evaluated in CML development (Weich et al., 2016). Little is known about the relation between GST polymorphisms and smoking associated with the risk of CML development, predicting treatment response, and clinical outcome simultaneously. Therefore, our overall aim of this work was to identify, for the first time, the correlation between GSTs genetic polymorphisms and CML cancer risk, treatment response and pattern its interactions with smoking as genetic modifiers in the etiology of CML disease in the Iranian population.

## 2 MATERIAL AND METHODS

### 2.1 Ethical compliance

Informed consent from patients, as well as from controls was obtained. The study was approved by Pasteur Institute of Iran Ethics Committee.

### 2.2 Study subjects

The study population was composed of 104 patients diagnosed with CML at Arad Hospital and Saba Oncology Clinic, and under treatment with TKI (Imatinib 400,800 mg/day) with a mean of follow up of 61.13 months, ranging from four to 216 months. All of the patients were in the chronic phase (CP). BCR‐ABL1 transcripts and total ABL1 as internal control were measured by quantitative real‐time polymerase chain reaction (qRT‐PCR), according to log reduction international scale (SI) as previously described (Cross., 2009; Müller et al., 2009). In addition, 104 sex‐ and age‐matched healthy unrelated individuals without history of cancer or other chronic diseases were recruited from Arad and Torfeh Hospitals. The smokers were defined as individuals who have smoked at least one package of cigarettes daily; nonsmokers were defined as individuals who have never smoked. The patient and control groups were Iranian, and had the same ethnicity. Demographic data of the studied subjects are shown in Table 1.

**Table 1:** Demographic characteristics of the CML patients and control group

	CML patients	Controls	*p*‐value
Number	104	104	
Sex [n (%)]			
Males	55 (52.9%)	57 (54.8%)	0.78
Females	49 (47.1%)	47 (45.2%)	
Age (years)			
*SD* ± Mean	15.1 ± 43.8	15.57 ± 44.99	0.57
Smoking status [n (%)]			
Smokers	57 (54.80%)	31 (29.80%)	0
Nonsmokers	47 (45.2%)	73 (70.2%)	

Abbreviation: *SD*, standard deviation.

### 2.3 Definition of treatment response

Response definitions include major cytogenetic response (MCyR), complete cytogenetic response (CCyR), major molecular response (MMR),complete molecular response (CMR), and also disease phases include chronic phase (CP), accelerate phase (AP), and blastic phase (BP) were defined according to European Leukemia Net (ELN) criteria (Baccarani et al., 2009, 2013, 2006). Optimal ELN response by cytogenetic and molecular responses were described previously (Baccarani et al., 2009).

### 2.4 Genotype analysis

Genomic DNA was extracted from EDTA peripheral blood using salting‐out method (Miller, Dykes, & Polesky, 1988)). A multiplex PCR was used for GSTT1 (NM_000853.3) and GSTM1 (NG_009246.1) amplification with β‐globin gene as an internal positive control. The primers used were described in Özten et al., 2012. The absence of PCR products for GSTM1 and GSTT1 (219 and 480 bp respectively) in the presence of β‐globin PCR product (268bp) indicated the null genotype for each. The genotyping of GSTP1 c.313 A>G polymorphism (NG_012075.1) was carried out by using the polymerase chain reaction/ restriction fragment length polymorphism (PCR‐RFLP) method with Alw26I restriction enzyme (Thermo Fisher). The primer sequence and the PCR reaction conditions for GSTP1 amplification were followed as defined previously (Soylemez, Akbas, Seyrek, Mutluhan, & Camdeviren, 2006)). The 436 bp PCR product of GSTP1 Ile105Val was digested into fragments of 222 bp and 107 bp which was indicative of homozygous mutant genotype (GG), the presence of three bands of 329 bp, 222 bp, and 107 bp indicated the heterozygous genotype (AG), and the appearance of two fragments at 329 bp and 107 bp represented the wild genotype (AA). To ensure quality control, a 10% random sample of the total was genotyped twice by two different persons; genotypes were 100% concordant.

### 2.5 Statistical analysis

To evaluate the differences in the distribution of demographic features between the cases and controls, we used chi‐square test (χ2 test) and *t* test to compare the mean of age. Hardy–Weinberg equilibrium was carried out by comparing the observed and expected genotype frequencies for the GSTP polymorphism using a χ2 test. The same test was used to compare the frequency of GSTs genotypes among cases and controls, and also to measure their influence on response to treatment. In addition, the odds ratios (ORs) and 95% confidence intervals (CIs) were calculated. In order to evaluate disease penetrance for GSTP1, the standard genetic models (recessive, dominant, and additive) were used. We considered just a recessive model for GSTM1 and GSTT1, because multiplex PCR does not differentiate between wild type and heterozygous genotype. The association between combined polymorphisms and CML risk was assessed using logistic regression analysis. Probabilities of achieving MMR and event‐ free survival (EFS) were calculated by the Kaplan–Meier method and compared by the log‐rank test. EFS was defined as the length of time after treatment that a person remains free of the following events: loss of CCyR and MMR, progression to AP/BP or death. All statistical consequences were made using two‐sided tests and values of *p* ≤ 0.05 were considered significant. Statistical analyses were carried out using R software, version 3.4.2 (R foundation for statistical computing, Vienna, Austria). To assess positive or negative interaction between gene–gene and gene–‐environment (smoking‐genetic polymorphism) as modifier factors on additive scale, we calculated ORs with 95% confidence limits, in addition to the relative excess risk due to interaction (RERI = OR11 − OR10 − OR01 + 1), synergy index for interaction (SI = OR11 − 1/(OR10 − 1) + (OR01 − 1) and the attributable proportion of the disease due to interaction (AP = RERI/OR11) (Jiang & VanderWeele, 2014; Khoury & Flanders, 1996; Walter & Holford, 1978). In these formulae, OR11 (also referred to as ORgg) refers to OR for disease among individuals carrying both susceptibility genotype and, among smokers with the susceptibility genotypes. OR10 (ORg(‐)) is OR for disease among individuals carrying one susceptibility and one wild type genotype and, among nonsmokers with the susceptibility genotypes. OR01 (OR(‐)g) refers to OR for disease among individuals carrying one wild type and one susceptibility genotype and, among smokers without the susceptibility genotypes. OR00 is supposed to be 1 or reference low‐risk group that refers to OR for disease among individuals without the susceptibility genotypes and nonsmokers.

## 3 RESULTS

### 3.1 Demographic features of the studied subjects

In the current study, 104 CML patients and 104 controls were evaluated. There was no significant difference among patients and controls regarding gender distribution and the mean of age (*p* > 0.05), but there was significant difference between the two group in terms of smoking status as higher percentage (54.8% vs. 29.8%) of patients was smokers than controls (*p* = 0) (Table 1).

### 3.2 GSTM1, GSTT1, and GSTP1 genotypes distribution and risk of CML cancer

The frequencies of GST genotypes are shown in Table 2. The frequencies of the GSTT1 null (GSTT1‐) genotype in CML patients and controls were similar (2.09% vs. 1%). In contrast, the frequency of GSTM1 null (GSTM1‐; null genotype has no enzyme activity) genotype in CML patients was significantly higher than controls (67.3% vs. 49%). To analysis the association between GST polymorphisms and CML risk, we used a recessive genetic model for GSTM1 and GSTT1 genes. Analysis of recessive model showed significant difference for the individual genotype of GSTM1 (*p* = 0.008; OR: 0.46; 95% CI: 0.26–0.82), this suggests that individual genotype of GSTM1 null is associated with CML development. There was no significant difference for the individual genotype of GSTT1 between the patients and controls (*p* = 0.31; OR: 0.32; 95% CI: 0.03–3.19). We used different genetic models for GSTP1 gene and showed a trend significance (*p* = 0.04), following an additive model (GG>AG>AA), this suggests 1.569 fold increased risk for GG (Table 2). GSTP1 polymorphism was in agreement with the Hardy–Weinberg equilibrium for patients and controls (*p* = 0.19; *p* = 0.73).

**Table 2:** Association analyses between individual GST polymorphisms and CML risk

Genes	Genotype/Allele	Patients N = 104(%)	Controls N = 104(%)	Genetic models	*p‐ value*	OR (95% CI)
GSTM1^a^	Present	34 (32.7%)	53 (51%)			
	Null	70 (67.3%)	51 (49%)	Recessive	**0.008**	0.46 (0.26–0.82)
GSTT1^b^	Present	101 (97.1%)	103 (99%)			
	Null	3 (2.09%)	1 (1%)	Recessive	0.31	0.32 (0.03–3.19)
GSTP1^c^	AA	54 (51.92%)	66 (63.5%)	Recessive	0.07	0.38 (0.13–1.14)
	AG	38 (36.54%)	33 (31.7%)	Dominant	0.09	0.62 (0.35–1.08)
	GG	12 (11.5%)	5 (4.8%)	Additive	**0.04**	1.569 (1.016–2.423)
	A	146 (70.19%)	165 (79.33%)			
	G	62 (29.81%)	43 (20.67%)	—	**0.03**	0.61 (0.39–0.96)

Bold indicate significant values. Abbreviations: OR, odds ratio; CI, confidence interval.

a GSTM1(GenBank accession number: NG_009246.1).

b GSTT1(NM_000853.3).

c GSTP1(NG_012075.1).

### 3.3 Gene–gene interactions for combinations of GSTs genotypes and CML cancer risk

We analyzed the joint effect of GST polymorphisms or interaction between genes by logistic regression to evaluate double combined genotypes with considering additive model for GSTP1 gene and recessive model for GSTT1, GSTM1 genes (Table 3). Frequencies of GSTM1 null/GSTT1 present, GSTM1 null/GSTP1 M* as well as GSTT1 present/GSTP1 M* genotype combinations were significantly increased in patients (66.34%, 41.35% and 48.08%) than the controls (48.07%, 18.27% and 35.58%, respectively). This shows that these combined genotypes are associated with CML risk (*p* = 0.004; ORg(‐):2.28; 95% CI: 1.29–4.04; *p* = 0.005; ORgg: 2.85; 95% CI: 1.36–5.97; *p* = 0.05; OR(‐)g: 1.75; 95% CI: 0.99–3.06). Joint ORs for gene–gene combinations and RERI for GSTs and CML risk compared with the referent group are presented in Table 3. ORs for GSTM1 (null and present) and GSTT1 null, GSTM1 (null and present) and GSTP1 AA as well as GSTT1 null and GSTP1 M*/AA were not associated with CML development. The RERI for GSTM1‐GSTP1 was more than zero indicating more than additive effect between these genes (RERI:2.32), so the interaction was positive or superadditive. The RERI for GSTT1‐ GSTP1 was negative or subadditive (RERI: −0.74).

**Table 3:** Gene–Gene interactions for different combinations of GSTM1, GSTT1, and GSTP1 genotypes and CML cancer risk

Genotype combination	Cases [n(%)] N = 104	Controls [n(%) N = 104	*p‐*value (χ^2^, df)		OR		95% CI
GSTM1^a^/GSTT1^b^							
Null/Null	1 (0.96)	1 (0.96)	(df = 1)	1	ORgg	1.65	0.1‐27.40
Null/Present	69 (66.34)	50 (48.07)	(8.2, 1)	0.004	ORg(‐)	2.28	1.29‐4.04
Present/Null	2 (1.92)	0 (0)	(df = 1)	0.15	OR(‐)g	—	—
Present/present	32 (30.77)	53 (50.96)	Ref		Ref	1	—
Additive Interaction (RERI)					RERI_OR_	0.37	
GSTM1/GSTP1^c^							
Null/M*	43 (41.35)	19 (18.27)	(7.891, 1)	0.005	ORgg	2.85	1.36‐5.97
Null/AA	27(25.96)	32 (30.77)	(0.027, 1)	0.86	ORg(‐)	1.06	0.51‐2.18
Present/ M*	7 (6.73)	19 (18.27)	(2.302, 1)	0.12	OR(‐)g	0.46	0.17‐1.26
Present/AA	27 (25.96)	34 (32.69)	Ref		Ref	1	—
Additive Interaction (RERI)					RERI_OR_	2.32	
GSTT1/GSTP1							
Null/ M*	0 (0)	1 (0.96)	(df = 1) 1	1	ORgg	—	—
Null/AA	3 (2.88)	0 (0)	(df = 1) 0.088	0.088	ORg(‐)	—	—
Present/ M*	50 (48.08)	37 (35.58)	(3.846,df = 1)		OR(‐)g	1.75	0.99‐3.06
Present/AA	51 (49.04)	66 (63.46)	Ref	0.05	Ref	1	—
Additive Interaction (RERI)					RERI_OR_	−0.749	

Abbreviations: OR: odds ratio; CI: confidence interval; RERI: relative excess risk due to interaction; Ref: reference low‐risk group; M*: AG and GG; Null and M*: susceptibility genotypes.

a GSTM1(NG_009246.1).

b GSTT1(NM_000853.3).

c GSTP1(NG_012075.1).

### 3.4 Gene–environment interactions between GSTs genotype and smoking for CML risk

Joint ORs, RERIOR, AP, and SI for gene‐smoking combinations and CML risk are presented in Table 4. The reference group: subjects unexposed to smoking and genetic risk as being 1.0, the ORs (ORge) evaluating the impact of joint exposure to smoking and GSTM1 null or GSTP1 M* genotype was significantly higher than the ORs (ORge) evaluating the impact of each factor in the lack of the other. The OR (ORge) estimating the impact of joint exposure to smoking and GSTT1 null was insignificantly higher than the effect of each factor alone. The proportion of CML cancer attributable to the interaction of smoking and GSTM1 null was 42% and was 39%, 13% for GSTT1 null and GSTP1 M*, respectively (Table 4). The RERI, the relative excess risk due to interaction of GSTM1‐Smoking, and GSTT1‐Smoking was more than zero suggesting more than additive effect between these genes and smoking risk ( RERI = 2.756; AP = 0.42; SI = 1.99; RERI = 1.26; AP = 0.39; SI = 2.39 respectively). The RERI for GSTP1‐Smoking was 0.58 indicating a positive interaction between this gene and smoking risk, but with less effect than GSTM1 and GSTT1.

**Table 4:** Gene–environment interactions for different combinations of GSTM1, GSTT1, and GSTP1 genotypes and CML cancer risk

Genotype	Smoking	Cases [n(%)] N = 104	Controls [n(%)] N = 104	(χ^2^, df)	*p‐value *	OR		95% CI
GSTM1^a^								
Null	+	38 (36.54)	14 (13.47)	(19.65, 1)	**0**	OR_ge_	6.51	2.57–15.38
Null	−	32 (30.77)	37 (35.57)	(3.54, 1)	0.06	OR_g_	2.07	0.96–4.46
Present	+	19 (18.27)	17 (16.34)	(4.84, 1)	**0.02**	OR_e_	2.68	1.10–6.52
Present	−	15 (14.42)	36 (34.62)	Ref		Ref	1	SI = 1.99
Additive Interaction (RERI)				AP = 0.423		RERI_OR_ = 2.75		
GSTT1^b^								
Null	+	2 (1.92)	1 (0.96)	(1)	0.56	OR_ge_	3.17	0.28–36.00
Null	−	1 (0.96)	0 (0)	(1)	0.39	OR_g_	—	—
Present	+	55 (52.88)	30 (28.85)	(13.46, 1)	**0**	OR_e_	2.91	1.63–5.18
Present	−	46 (44.24)	73 (70.19)	Ref		Ref	1	—
Additive Interaction (RERI)				AP = 0.39		RERI_OR_ = 1.26		SI=2.39
GSTP1^c^								
M*	+	27 (25.96)	12 (11.54)	(12.70, 1)	**0**	OR_ge_	4.40	1.90–10.19
M*	−	23 (22.11)	26 (25)	(2.1, 1)	0.14	OR_g_	1.73	0.822–3.65
AA	+	30 (28.86)	19 (18.27)	(8.8, 1)	**0.003**	OR_e_	3.09	1.45–6.588
AA	−	24 (23.07)	47 (45.19)	Ref		Ref	1	—
Additive Interaction (RERI)				AP = 0.13		RERI_OR_ = 0.58		SI = 1.20

Bold indicate significant values. Abbreviations: OR, odds ratio; CI, confidence interval; RERI, Relative excess risk due to interaction; Ref, Reference low risk group; AP, attributable proportion of the disease due to interaction; SI, synergy index for interaction; M*: AG, and GG; Null and M*, Susceptibility genotypes.

a GSTM1(NG_ 009246.1).

b GSTT1(NM_000853.3).

c GSTP1(NG_012075.1).

### 3.5 Evaluating of treatment response and clinical outcome

**Figure 1:**
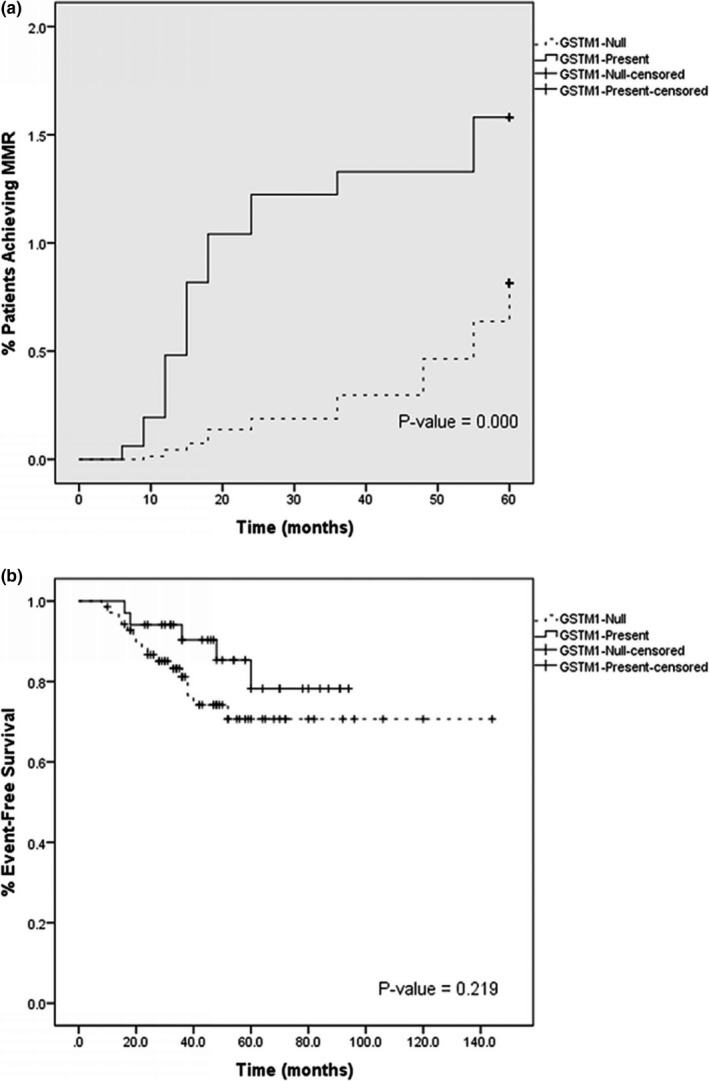
Kaplan–Meier plots: GSTM1‐null (dashed black line) versus GSTM1‐present (solid black line). (a) Probability to achieve major molecular response (MMR). (b) Probability of event‐free survival (EFS) during treatment (GSTM1; NG_009246.1)

In order to estimate the association between GSTs polymorphism and treatment response and clinical outcome, we calculated two endpoints by Kaplan–Meier plots for GSTM1. Patients with GSTM1‐null genotype had significantly a lower rate of MMR achievement compared to GSTM1present patients (*p* = 0.00) (Figure 1a). No association was detected for GSTT1 polymorphism. No significant difference was observed between patients with GSTM1‐null and GSTM1‐present genotype regarding EFS percentage (*p* = 0.21) (Figure 1b), and also no association was found for probability of EFS in GSTP1 genotype (data not shown). Furthermore, we compared different genotypes based on the achievement of optimal ELN response by cytogenetic and molecular responses at 3 and 6 months (Table 5). Patients with GSTM1/null genotype significantly had lower rate of MCyR and CCyR at 3 and 6 months (*p* = 0.01; *p* = 0.00 respectively), regarding molecular response, this difference was significant between patients with GSTM1/null and GSTM1/present at 3 and 6 months (*p* = 0.00) (Table 5). Patients with GSTP1AG/GG genotype had lower rate of CCyR at 6 month (*p* = 0.03). No significant difference was observed for MCyR rate at 3 month (*p* = 0.08). Patients with GSTP1 AG/GG genotype had inferior rate of molecular response at 3 and 6 months (*p* = 0.05; *p* = 0.009) (Table 5).

**Table 5:** Comparing of different genotypes based on the achievement of optimal ELN response at 3 and 6 months

Response		GSTM1^a^			GSTP1^b^		
		Null [n(%)] (N = 70)	Present [n(%) (N = 34)	*p‐ value*	AA [n(%)] (N = 54)	GG/AG [n(%)] (N = 50)	*p* value
Cytogenetic response^c^							
3 months	MCyR	10 (14.3)	14 (41.2)	0.017	15 (27.8)	7 (14)	0.086
	No MCyR	60 (85.7)	20 (58.8)		39 (72.2)	43 (86)	
6 months	CCyR	17 (24.3)	23 (67.65)	0.000	25 (46.3)	13 (26)	**0.032**
	No CCyR	53 (75.7)	11 (32.35)		29 (53.7)	37 (74)	
Molecular response^d^							
3 months	≤10%	2 (2.86)	10 (29.41)	0.000	20 (37.04)	10 (20)	**0.055**
	>10%	68 (97.14)	24 (70.59)		34 (62.96)	40 (80)	
6 months	<1%	6 (7.14)	18 (52.95)	0.000	25 (46.3)	11 (22)	0.009
	≥1%	(92.86)	16 (47.05)		29 (53.7)	39 (78)	

Bold indicate significant values.

a GSTM1(NG_009246.1).

b GSTP1(NG_012075.1).

c Optimal ELN response by cytogenetic response: Major cytogenetic response at 3 months (MCyR) or Complete cytogenetic response at 6 months (CCyR).

d Optimal ELN response by molecular response: BCR‐ABL1 ≤10% at 3 months or <1% at 6 months.

## 4 DISCUSSION

Although a number of studies have been carried out in different ethnic populations to evaluate the role of GSTs polymorphisms on CML susceptibility, but significance of association is still controversial. These inconsistencies might depend on geographic and ethnic differences among others (Weich et al., 2016). To our knowledge, no prior studies have been conducted regarding gene–gene and gene–environment interactions and their association with CML risk, drug response, and clinical outcome, especially the relationship between polymorphic chemical metabolizing genes and environmental carcinogens such as cigarette smoke. Thus, we report the impact of GST polymorphisms on CML risk and patients response in the first study from the Iranian population. In our study, a significant relationship was observed between risk to develop CML and GSTM1 and GSTP1 (on additive scale), indicating a meaningful association of these genes on CML susceptibility. These findings are consistent with previous association studies that demonstrated GSTM1/null and GSTP1/GG are predisposing factors to CML susceptibility (Bănescu et al., 2014; Kagita Sailaja, Rao, Rao, & Vishnupriya, 2010). Similar results with our findings were clearly described regarding increased risk of CML for the GSTM1/null genotype by Bhat et al. (2012), Lordelo et al. (2012) and Al‐Achkar et al. (2014). In contrast, several studies suggested that GSTM1 may not be predisposing factor for CML risk (Taspinar et al., 2008; Weich et al., 2016). In this study, no association was found between CML risk and GSTT1 polymorphism, consistent with Weich et al. (2016) report, this finding may be due to low frequency of individuals carrying the GSTT1‐null genotype or indicating a protective effect of GSTT1/null on CML risk. Contrary to our data, several studies have described increased risk of CML associated with the GSTT1/null genotype in different ethnicities (Özten et al., 2012; Taspinar et al., 2008). In the current study, we analyzed the relationship between dual combinations of gene–gene and gene–environment interactions using ORs as estimated effect in CML risk. Later, we analyzed gene–gene and gene–environment effect in CML risk using RERIOR in an additive model. The findings showed that GSTM1null and GSTP1M*(AG/GG) together [GSTM1‐null/GSTP1M*(AG/GG)] and with GSTT1 present are associated with CML development and increased risk especially for GSTM1‐null/ GSTP1M* genotype is superadditive. This finding is in agreement with other studies (Weich et al., 2016)). We carried out further analysis in order to determine the influence of simultaneous presence of GSTs genetic polymorphisms and smoking as an environmental risk factor that its role well has been fixed for lung cancer. Some attractive results were acquired; we showed a proportion of CML cancer attributable to interaction between smoking and GSTM1 null, GSTT1 null, and GSTP1M*genotypes around 42%, 39%, and 13% respectively, furthermore, the relative excess risk due to interaction was 2.75, 1.26, and 0.58 for GSTM1 null, GSTT1 null, and GSTP1M* genotypes, respectively. In the current study, a significant increase of 6.51‐, 3.17‐, and 4.40‐fold in the probability of having CML in individual who were both smokers and carried GSTM1/null, GSTT1/null and GSTP1M*genotypes, respectively, was indicated. Not many studies have evaluated gene–smoking interaction concerning CML risk to date, except, a recent study that did not find no significant association for susceptibility genotypes to CML risk when smoking was considered (Özten et al., 2012). In some studies CML risk has been associated with chemical exposures in the shoe‐making industry or benzene in Turkey (Yaris, Dikici, Akbulut, Yaris, & Sabuncu, 2004). Pharmacogenetic studies indicated that the detoxifying activity of GSTs enzymes keeps safe cells from the adverse effects of xenobiotics, but may modify drug efficacy in cancer cells, resulting in drug resistance (Kassogue et al., 2014). It is possible that therapy failure in the presence of the GSTM1/null and GSTP1/GG genotype may be related to pathways necessary for the activation of kinases to induce apoptosis (Weich et al., 2016). Therefore, we examined the role of GSTs genetic polymorphisms on TKIs response. Our data suggest that patients with GSTM1‐null and GSTP1 AG/GG genotypes have significantly a lower rate of MMR achievement, and also have a lower rate of CCyR at 6 months. No association was found for GSTT1. This finding concerning GSTM1 null genotype is consistent with a recent study that GSTM1/null was associated with Imatinib (Gleevec) failure (Davies et al., 2014). In contrast, the recent report by Weich et al. (2016) showed a lower rate of MMR and EFS for GSTM1/present patients, and also indicated treatment failure for GSTP1/GG genotype compared to GSTP1/ AA + AG genotype (Weich et al., 2016). Regarding probability of EFS during treatment, no association was found in patients with GSTM1/null and GSTP1M* genotypes compared to GSTM1/present and GSTP1/ AA genotypes. These findings are not agreement with report of Weich et al. (2016). In conclusion, our study suggests the influence of GSTM1 and GSTP1 polymorphisms on CML risk and treatment response, and also the interaction between GSTs polymorphisms and smoking plays a significant role on CML susceptibility.

## CONFLICT OF INTEREST

The authors declare that they have no conflict of interest.

## AUTHOR CONTRIBUTIONS

GR: performed the experiments, analyzed the data, and wrote manuscript, DA: assisted in final revision of manuscript, FG: performed the experiments, MH: designed the study and assisted in revision of manuscript, AK: assisted in analysis and interpretation of data, HJ: provided the CML samples and clinical data, and revised manuscript, RAK: assisted in collection of CML samples and clinical data, and all authors reviewed and provided final approval for the paper.
